# Editorial for the Special Issue “Advances in Molecular Biology Methods in Hepatology Research”

**DOI:** 10.3390/cimb47080578

**Published:** 2025-07-22

**Authors:** Dileep G. Nair, Ralf Weiskirchen

**Affiliations:** Institute of Molecular Pathobiochemistry, Experimental Gene Therapy and Clinical Chemistry (IFMPEGKC), RWTH University Hospital Aachen, D-52074 Aachen, Germany; grdileep@gmail.com

We are delighted to present this Special Issue of *Current Issues in Molecular Biology*, entitled “Advances in Molecular Biology Methods in Hepatology Research.” This collection of ten articles, including five original research papers and five reviews, showcases important technical and conceptual innovations that are poised to shape the future of hepatology. The contributions come from eight countries (Belarus, China, Germany, Italy, Japan, Malaysia, Serbia, and Taiwan), reflecting the truly global and collaborative nature of contemporary hepatology research.

Innovations in molecular biology methods and diagnostics have long underpinned major advances in understanding liver pathophysiology [1,2]. With the increasing complexity and global incidence of liver diseases such as metabolic-dysfunction-associated steatohepatitis (MASH), viral hepatitis, and hepatocellular carcinoma (HCC), it is clear that highly refined and specialized techniques are necessary to dissect the biochemical and cellular underpinnings of these conditions and to identify new molecular markers [3,4]. The research featured in this Special Issue highlights new and refined tools or findings that accelerate our ability to capture and interpret the intricacies of liver function and disease.

The five original research papers break new ground by applying state-of-the-art methodologies to diverse aspects of hepatology. In the first study, patients with EGFR-mutated NSCLC showed a median overall survival of 13 months, whether or not they had liver metastasis, though 9-year survival rates were higher (12.5%) among those with metastases than those without (3.4%) [5]. The second article investigated IL-28B rs8099917 genotypes in HCV patients after liver transplantation, revealing that the IL-28B genotype TT correlated with higher miRNA-122 levels, better outcomes, and a lower rate of acute cellular rejection [6]. In the third study, aspirin reduced lipopolysaccharide-induced liver inflammation and thrombus formation in mice, emphasizing its anti-inflammatory and antiplatelet effects [7]. The fourth article showed that lipid-nanoparticle-delivered siRNA targeting Cyp2e1 alleviated acetaminophen-induced liver injury by modulating PPAR-related pathways and reducing oxidative stress [8]. Lastly, the fifth study identified HBV genotype D as predominant in Belarus, with occasional introductions of other variants, and underscored how local virus circulation fuels the ongoing hepatitis B epidemic (contribution 1).

The five review articles critically evaluate how advancements in molecular biology can effectively be translated into clinical and translational research. They explore emerging techniques in genomics, proteomics, and metabolomics specific to hepatology, providing insights into their potential to lead to breakthroughs in patient stratification, biomarker discovery, and tailored treatment modalities. These reviews highlight the promise of next-generation sequencing platforms, innovative in vitro models, and artificial-intelligence-driven data analysis as essential tools for understanding disease pathways across the spectrum of liver disorders. The first review analyzes how herbal preparations can have hepatoprotective effects by modulating the gut microbiota and improving liver pathology in conditions such as MASLD, MASH, and hepatic steatosis (contribution 2). The second review discusses the challenges of treating NSCLC patients with liver metastases due to the liver’s immunotolerant environment, exploring combination therapies, immunotherapies, and surgical resection as potential strategies (contribution 3). In the third review, advanced in vitro systems, particularly organ-on-a-chip platforms, are highlighted as promising tools for predicting drug-induced liver injury and reducing the cost and duration of drug development (contribution 4). The fourth review focuses on lipopolysaccharide (LPS)-mediated liver damage, noting how TLR4-driven inflammation leads to hepatic injury and how agents such as aspirin could mitigate these effects (contribution 5). Finally, the fifth review addresses the role of simple sugars in exacerbating liver disease through inflammation, de novo lipogenesis, oxidative stress, and dysbiosis, pointing to both lifestyle and molecular interventions for prevention and treatment (contribution 6).

By assembling this Special Issue, we aim to highlight the ongoing evolution of molecular biology approaches in addressing the most pressing challenges in hepatology of today. The methods described here have significantly expanded our toolbox, opening up new avenues not only for basic scientists but also for clinicians striving to translate laboratory discoveries into improved patient outcomes. We believe that these advances collectively represent a step forward for the field, propelling hepatology research towards more precise, patient-oriented strategies.

We hope that the diverse perspectives and methodological innovations showcased in this Special Issue will be both stimulating and valuable to our readers, inspiring collaborative efforts to advance the field of molecular biology in hepatology research.

As interest in this field continues to grow, we are pleased to announce a second edition dedicated to “Advances in Molecular Biology Methods in Hepatology Research.” We invite clinicians and researchers worldwide to submit their latest findings in the forms of original research, comprehensive reviews, interesting case reports, perspectives, and other relevant manuscripts. We look forward to creating an even broader platform for knowledge exchange, fostering collaborations that will drive the field of hepatology research forward.

## Data Availability

No new data were created or analyzed in this study. Data sharing is not applicable to this article.
